# First tagging data on large Atlantic bluefin tuna returning to Nordic waters suggest repeated behaviour and skipped spawning

**DOI:** 10.1038/s41598-022-15819-x

**Published:** 2022-07-11

**Authors:** Kim Aarestrup, Henrik Baktoft, Kim Birnie-Gauvin, Andreas Sundelöf, Massimiliano Cardinale, Gemma Quilez-Badia, Iñigo Onandia, Michele Casini, Einar Eg Nielsen, Anders Koed, Francisco Alemany, Brian R. MacKenzie

**Affiliations:** 1 National Institute of Aquatic Resources, Technical University of Denmark, Silkeborg, Denmark; 2 Department of Aquatic Resources, Institute of Marine Research, Swedish University of Agricultural Sciences, Lysekil, Sweden; 3 La Conillera Gran, 8 Castellví de la Marca, 08732 Barcelona, Spain; 4 AZTI, Marine Research, Basque Research and Technology Alliance (BRTA), Sukarrieta, Spain; 5 Department of Biological, Geological and Environmental Sciences, University of Bologna, Bologna, Italy; 6 International Commission for the Conservation of Atlantic Tunas, GBYP, Madrid, Spain

**Keywords:** Animal migration, Behavioural ecology, Conservation biology, Population dynamics

## Abstract

Atlantic bluefin tuna (*Thunnus thynnus;* ABFT) is one of the most iconic fish species in the world. Recently, after being very rare for more than half a century, large bluefin tunas have returned to Nordic waters in late summer and autumn, marking the return of the largest predatory fish in Nordic waters. By tagging 18 bluefin tunas with electronic tags (pop-up satellite archival tags), we show that bluefin tuna observed in Nordic waters undertake different migration routes, with individuals migrating into the western Atlantic Ocean, while others stay exclusively in the eastern Atlantic and enter the Mediterranean Sea to spawn. We additionally present evidence of possible skipped spawning inferred from behavioural analyses. In Nordic waters, ABFT are primarily using the upper water column, likely reflecting feeding activity. The results support the hypothesis that ABFT migrating to Nordic waters return to the same general feeding area within the region on an annual basis. These observations may have important implications for management because (1) tunas that come into Nordic waters might represent only a few year classes (as evidenced by a narrow size range), and thus may be particularly vulnerable to area-specific exploitation, and (2) challenge the assumption of consecutive spawning in adult Atlantic bluefin tuna, as used in current stock assessment models. Without careful management and limited exploitation of this part of the ABFT population, the species’ return to Nordic waters could be short-lived.

## Introduction

Atlantic bluefin tuna (*Thunnus thynnus,* ABFT) is one of the most iconic fish species in the world, with interest dating as far back as Aristotle^1^. ABFT is a fascinating species due to its large size, its endothermic system, which allows for very long migrations between cold and warm waters^2,3^, and its high food value, recently exemplified by high prices in the Japanese sushi market. Like many other economically valuable iconic species, it has also been subject to intense fishing pressure in recent decades, resulting in over-exploitation^4^ and a precipitous drop in the population size, necessitating important conservation and management actions. ABFT consist of at least two genetically distinct populations^5^: one spawning in the Mediterranean Sea (Eastern stock)^6^, and one in the Gulf of Mexico (Western stock)^7^, with individuals from both populations mixing in the northeast Atlantic^8–11^. Recent evidence also suggests additional spawning grounds may be present in the North Atlantic^12^, like in the Slope Sea of northeast United States for example, though whether it is a separate population remains unclear^13^.

ABFT is managed through the International Commission for the Conservation of Atlantic Tunas (ICCAT). ICCAT divides the Eastern and Western stocks at the 45° W, meridian, and historically assumes no mixing occurs across this boundary^14^. This division is viewed more as a pragmatic measure, as both populations have been shown to cross the Atlantic Ocean and therefore mix. ABFT also regularly migrated to Scandinavian waters, with observations documented as far back in time as the Stone Age^15^, typically present from mid/late summer until late autumn^16–18^. The species’ presence in Scandinavian waters in summer and autumn created a highly-prized commercial and recreational fishery, especially in the Øresund Strait between Denmark and Sweden^19^. The plight that caused a severe decline in ABFT also affected individuals migrating to Scandinavian waters, causing catches of ABFT in Denmark and Sweden to drop to almost zero in the early 1960s. Unfortunately, this happened before the development of electronic tagging and genetic methods, so knowledge of these fish mainly consists of commercial and recreational catch records as summarized in the literature (e. g., ICES Bluefin Tuna working group reports)^20^.

Following a continued decline of the species, ICCAT instated a recovery plan for the Eastern stock in 2007^21^ to reverse the population decrease, which included, among other technical measures, restricting annual total allowable catches and increasing minimum landing size. Consecutive updates of this recovery plan (2008 and 2010) decreased the total catch of ABFT and resulted in positive stock development described by the 2014 stock assessment^22^. A continued positive development has since been observed, resulting in a concurrent increase in the total allowable catch defined by ICCAT. The quick increase in biomass of the Eastern stock was also favoured by the extraordinary recruitment of the 2003 cohort, probably associated with a worldwide intense heat wave that strongly increased the Western and Central Mediterranean sea surface temperatures during July 2003. This warming may have resulted in better larval survival and, and thus a higher recruitment^23^. Following the successful development of the recovery plan, ABFT reappeared west of Ireland^24^ and in waters near the southwest of the United Kingdom^25^. More recently, sightings of ABFT in the Skagerrak-Kattegat area have increased, and sparked great interest (both by scientists and the public) in studying the largest predatory fish in the area.

The use of electronic tagging to study aquatic species has seen a large increase in recent years because it enables researchers to address key questions about movements and behaviours that otherwise could not be answered^26^. Given that such tools were not available when ABFT were previously abundant in the region, the behaviour and habitat use of the species in the area remains largely unknown. Our aim was to deploy pop-up satellite archival tags (PSATs) on specimen caught by experienced big game fishermen in this area, newly retaken by the species. The relatively unique nature of this ecological event (i.e., re-discovery and use of a former habitat after many decades of rarity by a highly mobile top predator species) could provide unique insights to how such predators learn and establish new migration behaviours as population size changes, and under changing ecosystem conditions. Within the framework of an ICCAT GBYP program initiative, the first study was undertaken in 2017, where our specific objectives were to (1) map the horizontal ocean migration patterns of individual ABFT that visit Scandinavian waters, (2) broadly describe their behaviour in terms of migration routes, diving frequency, as well as depth and temperature range utilization, and (3) test whether ABFT that visit Nordic waters return the following year.

## Materials and methods

### Capture

ABFTs were captured by experienced volunteer big game anglers using rod and reel. Because the ABFTs historically caught by anglers in the region have been large (typically > 2 m)^18,20^, and because all fish had to be captured, tagged and released in good condition, there were very strict requirements for the angling teams to follow. To be selected, fishing teams had to provide an appropriate boat (e.g. including VHF and safety equipment), powerful gear (i.e. minimum 130 lbs main line and 180 lbs leader, non-stainless steel circle hooks), and documented experience with big game fishing of large ABFT or species of similar size. Participating boats had to be of a size that permits safe operation of the fishing gear in open sea conditions between Denmark and Sweden. In total, 53 individual boats and crews participated in the fishing in 2017.

**Figure 1:**
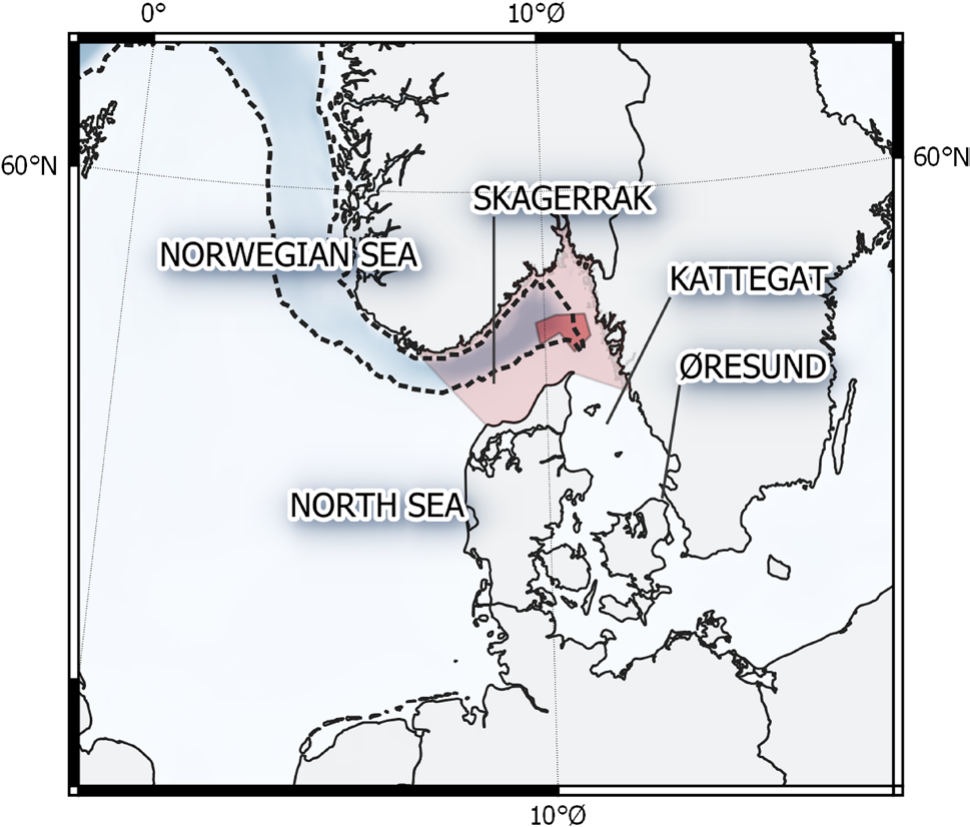
Map showing the seas around the Nordic countries. Dark red area shows catch and tagging area, larger light red areas denotes ICES Area 3a. The Norwegian Trench is the darker blue band following the Norwegian coastline. Map created with QGIS 3.14 (https://www.qgis.org).

Fishing was performed over eight days, depending on weather conditions, during the period 8–24 September 2017 in the central Skagerrak (see Fig. 1). The fishing was done typically drifting, using balloons and baited hooks. Baits were generally mackerel (*Scomber scombrus*), though garfish (*Belone belone*) and herring (*Clupea harengus*) were sometimes used. Some teams used chumming, i.e. throwing fish parts and blood in the water to attract the tuna. Each boat had 2–10 crew members on-board. The fishing area was restricted to around eight nautical miles from a predefined position, where one of two tagging boats were placed (herein referred to as the Danish and Swedish tagging boats), such that a tagging boat could reach any fishing boat within 20 min.

### Tagging and sampling operations

Due to national legal restrictions, the Swedish tagging boat could only tag fish caught by fishing teams departing from Swedish harbours. The Danish tagging boat operated under no such restrictions. In addition, tagging procedures differed between the two tagging boats.

For the Danish tagging boat, when a tuna was hooked, the tagging boat was contacted and immediately moved toward the location of the fishing boat. Once at the fishing boat, the tuna was carefully gaffed in the very front of the lower jaw (where there is minimal chance of damage to the fish), and subsequently towed at 2–3 knots behind the boat. Gaffing is a common method used to tag handle and tag billfishes and is approved by ICCAT. All gaffs had two ropes tied to them, where one end was handed over to the tagging boat for transferring, and the other one was maintained on the fishing boat, until the fish was secured by the tagging team. The fishing boat then released the second rope. The fish was first swum behind the tagging boat to assess its condition (movement, colours, ventilation, tail beats). If and when the fish’s condition was deemed acceptable, the fish was pulled on-board the boat using a winch system (note that no fish were released untagged due to poor condition). While out of the water, the fish was ventilated with fresh sea water using a large water hose connected to an on-board pump. On-board, the fish was kept on a mat tailored specifically for tagging of large pelagic fish, and its eyes were covered with a large wet towel to keep the fish calm. The tunas were first tagged with a pop-up satellite archival tag (Wildlife Computers MiniPAT 348F, hereafter ‘tags’) using a 15 cm tether fitted with a large Domeier dart at the second dorsal fin (as detailed in Ref.^7^), and then tagged with a conventional ICCAT spaghetti tag, posterior to the PSAT position. In the meantime, two other tagging team members removed the hook (when possible) and took a small fin clip sample (< 0.5 cm). The fish were then measured for curved fork length (CFL) and released back into the water. Generally, the procedure was done within 3 min. After tagging, the fish was swum behind the boat and its behaviour was observed to ensure a good condition. Once deemed fit for release, the tagging boat engine was stopped, the gaff was removed, and the fish released. One fish could not be tagged on-board, because a different tagging boat was used that day, and its design prevented hauling the tuna on-board. Instead, it was tagged in the water alongside the boat. All methods were carried out in accordance with relevant guidelines and regulations following approved ICCAT GBYP protocols regarding fish care and handling during tagging operations (https://www.iccat.int/GBYP/Docs/Tagging_Manual.pdf). Further, all procedures and experimental protocols were conducted in accordance with the Danish Experimental Animal Inspectorate permit for DTU (License no. 2017-15-0201-01164) and approved by DTU Aquas’ animal welfare committee. The reporting follows the recommendations in the ARRIVE guidelines (https://arriveguidelines.org/).

A similar procedure was used for the Swedish tagging boat, though all fish were tagged in the water, alongside the boat. Once a tuna was hooked, the tagging boat was called and moved to the site of the fishing boat. An experienced tagger was then transferred onto the anglers’ boat, where the fish was gaffed, and tagged alongside the anglers’ boat. A 60 cm pole, equipped with a 15 cm tether and large Domeier dart, was used to anchor the tag at the second dorsal fin. A small fin clip was obtained, and the hook removed whenever possible. The boat was continuously moving during the procedure, to ensure sufficient ventilation. The fish was then towed behind the boat, at a speed between 1 and 2 knots. When the signs of recovery were evident (strong coloration, tail beats and head movement), the engine was stopped and the fish was released from the gaff. Again, all the tags were deployed following approved ICCAT GBYP protocols regarding fish care and handling.

All PSATs were programmed to detach from the fish after 365 days. These tags recorded light, pressure (depth) and temperature every 5 s. After detachment, tags were programmed to transmit subset data packages every 60 s (see Wildlife Computers for details). All tags were programmed to release from the study animal and start transmitting data if they remained 3 days at a constant depth. This could occur if an animal died, or if a tag detached prematurely in which case the tag would be at a constant depth at the surface.

### Data processing

All fish were genetically assigned to either the Mediterranean (MED) or the Gulf of Mexico (GOM) populations with a highly discriminatory panel of Single Nucleotide Polymorphisms (SNPs)^11,27^ (Table 1).

**Table 1:** Data and summary statistics on tagged Atlantic bluefin tuna tagged in Skagerrak in 2017.

Tag ID	CFL (cm)	Origin	Tag date	Tag position (lat, long)	Detach date	Detach position (lat, long)	Detach reason	Days at large	Total track length (km)	Temperature (°C) Area 3a	Depth Area (m) 3a	Exit Area 3a	Days in Area 3a	Tagging strategy	Tag team
162993	227	MED	Sep 09	58.09, 10.80	Oct 07, 2017	59.27, 5.17	Premature	28	1252	15.8 (15.4–16.4)	18 (1–188)	Sep 17	8	In water	Sweden
162995	240	MED	Sep 15	58.04, 10.88	Oct 02, 2017	58.41, 9.10	Premature	17	925	15.2 (12.5–16.2)	11.6 (0–328)	Oct 15	30	In water	Sweden
162997	235	MED	Sep 15	58.05, 10.90	Oct 22, 2017	59.43, 4.70	Premature	37	1324	15.1 (12.7–16.6)	22.5 (0–216)	Oct 16	31	In water	Sweden
163000	225	MED	Sep 15	58.09, 10.96	Oct 26, 2017	58.41, − 7.67	Pin broke	41	1850	14.5 (12.7–15.9)	23.1 (0–320)	Oct 18	33	In water	Sweden
163001	256	MED	Sep 16	58.06, 10.82	Oct 16, 2017	60.96, 4.00	Premature	30	1808	15.1 (12.1–16.5)	16.3 (0–264)	Oct 06	20	In water	Sweden
163002	215	MED	Sep 16	58.08, 10.83	Oct 07, 2017	58.38, 1.93	Premature	21	1545	15.3 (14.8–15.8)	15 (0–68)	Sep 18	2	In water	Sweden
163004	240	MED	Sep 22	58.14, 11.02	Oct 31, 2017	58.62, 4.91	Premature	39	1819	14.2 (12.4–15.8)	28.2 (0–520)	Oct 24	32 days	In water	Sweden
163005	239	MED	Sep 22	58.03, 10.75	Oct 09, 2017	62.41, 3.75	Premature	17	1486	15.2 (13–15.9)	17.1 (0–464)	Sep 30	8 days	In water	Sweden
34840	247	MED	Sep 09	MED	Sep 03, 2018	58.43, 10.58	Pin broke	359	22,268	14.8 (12.6–16.5)	20.1 (0–172)	Oct 17	38 days	Onboard	Denmark
34859	246	MED	Sep 18	58.01, 10.78	Sep 18, 2018	57.77, 4.57	Completed	365	25,937	15 (14.6–15.5)	NA (NA–NA)	NA	NA days	Onboard	Denmark
34861	221	MED	Sep 23	58.03, 10.65	Apr 27, 2018	42.14, -51.63 MED	Pin broke	216	16,002	14.5 (12.2–15.8)	23.4 (0–216)	Oct 15	22 days	In water	Denmark
162996	230	MED	Sep 15	58.06, 10.65	Sep 18, 2017	58.05, 11.15	Premature	3	NA	NA (NA–NA)	NA (NA–NA)	NA	NA days	In water	Sweden
163003	227	MED	Sep 21	58.12, 10.96	Sep 24, 2017	58.18, 10.89	Premature	3	NA	NA (NA–NA)	NA (NA–NA)	NA	NA days	In water	Sweden
34839	251	MED	Sep 09	58.04, 10.67	Sep 12, 2017	58.04, 10.62	Premature	3	NA	NA (NA–NA)	NA (NA–NA)	NA	NA days	Onboard	Denmark
162992	225	MED	Sep 09	58.17, 11.01	NA	NA;NA	NA	NA	NA	NA (NA–NA)	NA (NA–NA)	NA	NA days	In water	Sweden
162994	185	MED	Sep 09	58.08, 10.88	NA	NA;NA	NA	NA	NA	NA (NA–NA)	NA (NA–NA)	NA	NA days	In water	Sweden
162998	230	MED	Sep 15	58.03, 10.90	NA	NA;NA	NA	NA	NA	NA (NA–NA)	NA (NA–NA)	NA	NA days	In water	Sweden
162999	245	GOM	Sep 15	58.05, 10.92	Dec 22, 2017	64.98, 10.77	Premature	98	NA	NA (NA–NA)	NA (NA–NA)	NA	NA days	In water	Sweden

Origin refer to the genetic assignment to either the Mediterranean (MED) or Gulf of Mexico (GOM) populations. Premature detachment refers to tags surfacing and initiating transmission before the programmed date for unknown reasons. Pin broke refers to the release pin breaking without the release mechanism initiated (e.g. due to large stress on the pin). Completed means tag initiated release on programmed time. NA indicates data that is not available due to poor data transmission, or tags that never transmitted. Range is provided in brackets.

Tracks for each fish were estimated using the Global Position Estimator 3 model (GPE3; Wildlife Computers). The model compares depths, light levels and temperatures recorded by the tags to reference data on bathymetry (ETOPO1-Bedrock), times of daily twilight, and sea surface temperature (NOAA OI SST, www.esrl.noaa.gov/psd)^28^. Based on these data and a user-defined assumed movement speed model, the model reconstructs the most probable trajectory given the data and model. We assigned animal maximum movement speed = 5 m s^−1^ for all fish as preliminary model runs indicated that assigning lower speeds constrained the fish movements and resulted in worse overall model fit. This is also in line with the GPE3 user guide, which suggests to input movement speed “on the high side” (https://wildlifecomputers.com/blog/using-gpe3-to-improve-geolocation-estimates/). A previous study using similar tags reported mean positional errors of 1.2 ± 0.1° latitude and 1.5 ± 0.1° longitude^25^. Total track lengths were estimated based on daily average positions (Table 1).

**Figure 2:**
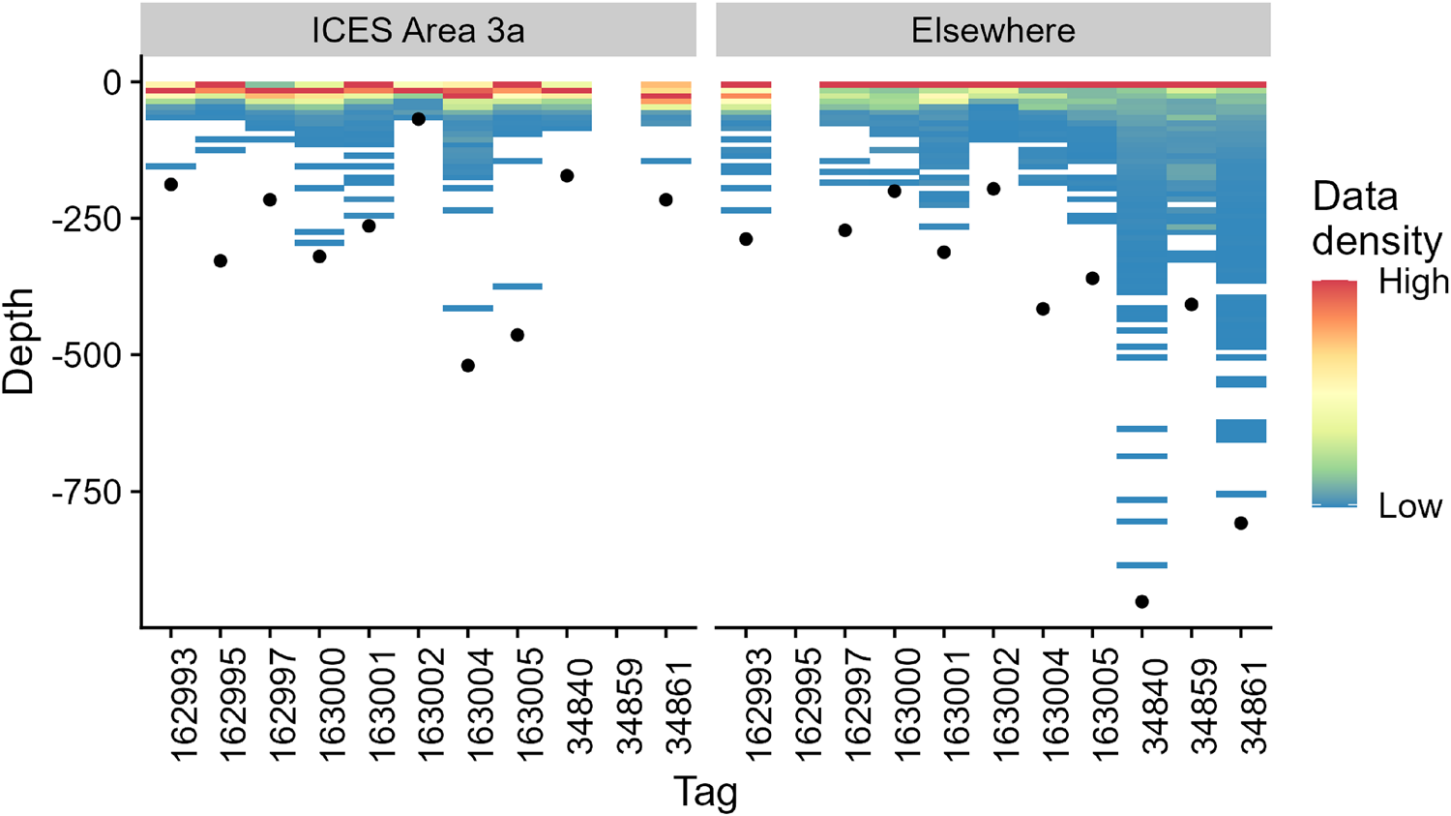
Distribution of registered depth while inside ICES Area 3a (Skagerrak, see Fig. 1, left) and for the three long tracks reaching into the Atlantic (right). Each column represents one fish, with the bar colour indicating the relative density of observed swimming depths. Black dots indicate maximum depth values registered by the tag.

To facilitate the emphasis on behaviour in Nordic waters and because few fish retained the tag for extended periods, we segregated the data based on the estimated trajectories of each fish into two periods: (i) while residing in Nordic waters after tagging, defined by estimated positions as, being within ICES Area 3a and (ii) during the remainder of the track (Fig. 2). To ensure comparability between all tagged fish, only data received through satellite transmissions were used in these analyses.

Two tags were recovered, which detached from tagged tuna 359 and 365 days post-tagging. Thus, two full-year datasets were available from these recovered tags. These datasets were explored in detail for signs of spawning activity using visualization *sensu*^29^. More specifically the data were searched for patterns consisting of frequent and brief oscillatory movements up and down through the mixed layer to sub-thermocline waters at night^30^, resulting in thermal profiles characterized by oscillations around the thermocline. These data were matched with water temperatures previously recorded during ABFT spawning^31^.

## Results

### Tagging and tag retention

In total, 18 ABFT (Table 1) were tagged and sampled. The tagged ABFT were likely all adults based on ICCAT length-at-maturity relationships^2^, and ranged in size from 185 to 256 cm curved fork length (mean ± SE = 232 ± 3.8), with the vast majority between 220 and 250 cm. Three tags popped approximately 3-days post-tagging, likely reflecting mortality. Three tags never transmitted a location or data, perhaps because (1) they popped in locations with poor satellite coverage, (2) the tags malfunctioned, (3) the antennas were damaged, or (4) a mortality event occurred and the tag was somehow prevented from surfacing. Given previous evidence, it is more likely that the tags malfunctioned or the antennas were damaged than mortality events. It is not uncommon for tags to fail to report^32,33^. Of the remaining 12 tags, nine popped within four months, one after seven months, and two after 359 and 365 days, respectively. One tag that surfaced within 4 months transmitted too little data to enable track estimation and was omitted from the analyses. The remaining 11 tags yielded a total of 1277 days of useable data. Both tags that popped after 359 and 365 days surfaced in the Skagerrak area, and were recovered at sea, providing access to the full archived dataset (temporal resolution 5 s) rather than transmitted data only. All 11 fish were genetically assigned to the Mediterranean population.

### Depth and temperature use within ICES Area 3a

While in ICES Area 3a, the fish were surface oriented with on average 21% (min–max: 4–43%) of all observations in the top 10 m of the water column, and 60% (min–max: 50–74%) of all observations in 10–100 m. Individual maximum depth ranged from 68 to 520 m (Fig. 2, left panel). The temperature experienced by the fish in Area 3a ranged from 7.1 to 17.0 °C. Average sea surface temperature in ICES Area 3a decreased from approximately 16.1 °C (min–max: 15.6–16.6) at the start of tagging (9 September 2017) to approximately 12.9 °C (min–max: 11.2–14.1) when the last tagged fish left the area (24 October 2017) (data obtained from E.U. Copernicus Marine Service Information, https://doi.org/10.48670/moi-00018). The three ABFT with much longer tracks had a larger variation in depth use (Fig. 2, right panel).

### Migration routes

**Figure 3:**
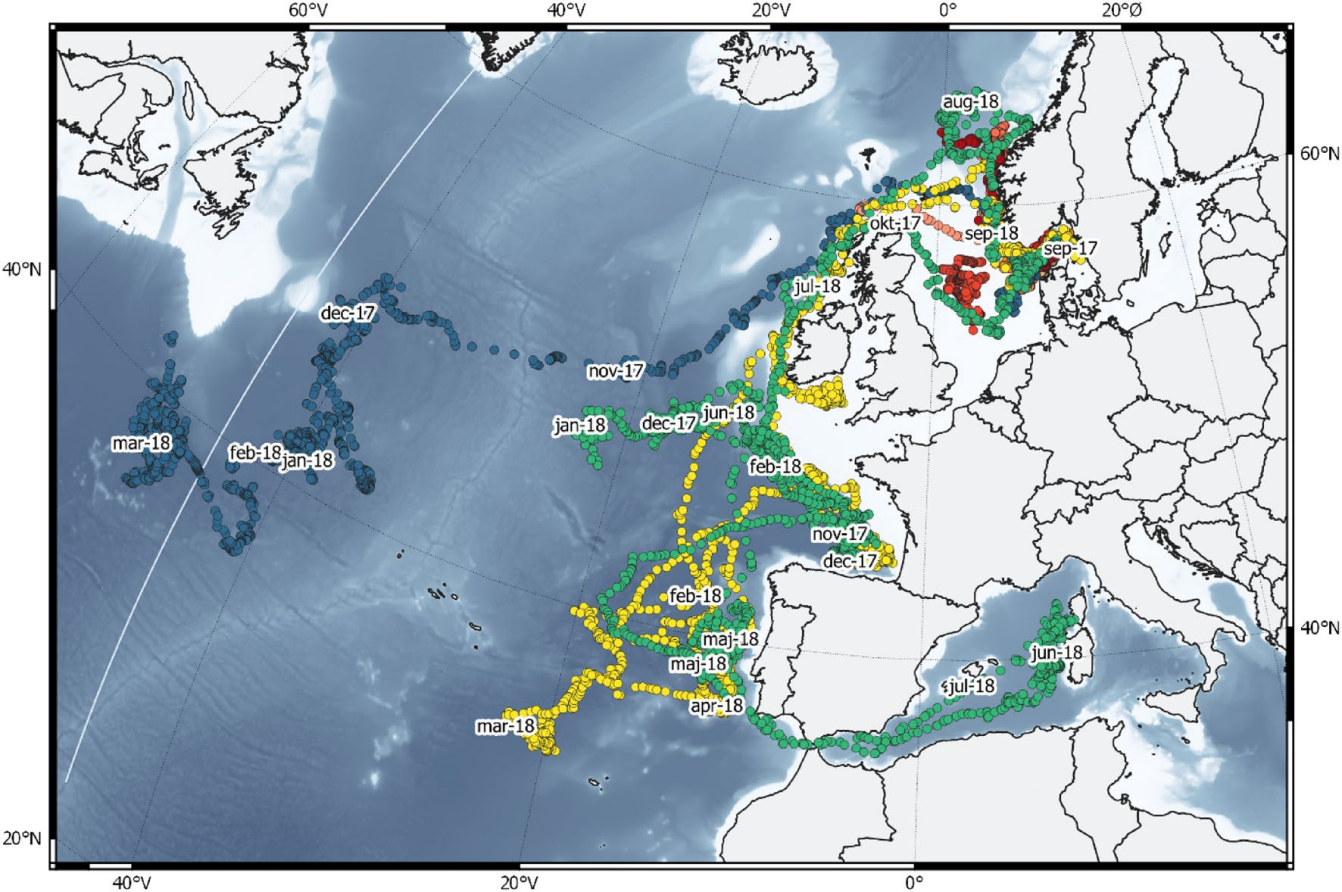
Estimated trajectories and pop-off locations for the 11 fish that yielded usable data. Trajectories of three fish with tag attached for more than seven months are coloured in blue (34861), yellow (34840) and green (34859). Trajectories of the remaining eight fish are coloured in shades of red. On each track mid-month positions are shown denoted by month-y. White dashed line indicates the ICCAT stock delimitation 45°W meridian. Tagging area (white rectangle), ICES Area 3a (green polygon) and surface pop-off positions of the two longest deployments shown in inserted map. Map created with QGIS 3.14 (https://www.qgis.org).

Modelled trajectories are shown in Fig. 3. The tagged ABFT moved out of the Skagerrak (ICES Area 3a) within 2 to 38 days after tagging, with the last fish leaving 24 October 2017. Most fish followed the Norwegian Trench (the darker coloured band following the Norwegian coast in Fig. 1), moving north along the Norwegian coast before heading west across the northern North Sea or southern Norwegian Sea, passing north of the British Isles in a narrow band between Scotland and the Orkney Islands, and then turning south and southwest. Of the three tags that remained on the fish for seven months or more, one (ID 34861) continued southwest/west, and headed towards the continental shelf break around the Flemish Cap off southern Newfoundland (Canada), moving south just east of the Grand Banks, before turning west and passing the 45° Meridian on 3 March 3 2018. The tag then surfaced prematurely on 25 April 2018, south of Newfoundland.

The two remaining fish (ID 34840 and 34859), for which the tags were recovered in Nordic waters approximately one year after tagging, showed similar routes to each other for a large part of the time spent in the Atlantic. Both fish left the Norwegian Sea in October, continued south past Ireland, turning southeast and entering the Bay of Biscay in November. Fish 34840 stayed in the Bay of Biscay for an extended period and swam southwest/west to an area west of Southern Portugal and the Gibraltar Strait in mid-February. The fish stayed in the area until mid-June, before moving back up north towards Ireland, within a few weeks, staying exclusively over the continental shelf. The fish then moved past the British Isles and headed east, straight back into Skagerrak where the tag surfaced after 359 days. From the Bay of Biscay, fish 38459 moved northwest close to the mid-Atlantic ridge in mid-November before returning to the Bay of Biscay in mid-February. In mid-March, it made a similar movement to fish 34840, and headed south to an area west of Southern Portugal and Gibraltar Strait. The fish then entered the Mediterranean Sea through the Gibraltar Strait on 22 May 2018. It then moved quickly east to the west coasts of Corsica and Sardinia, before turning back and leaving the Mediterranean Sea on 20 July 2018. The fish then moved quickly north past the British Isles, heading north east and then South where it surfaced close to the Skagerrak after 365 days.

### Spawning behaviour

**Figure 4:**
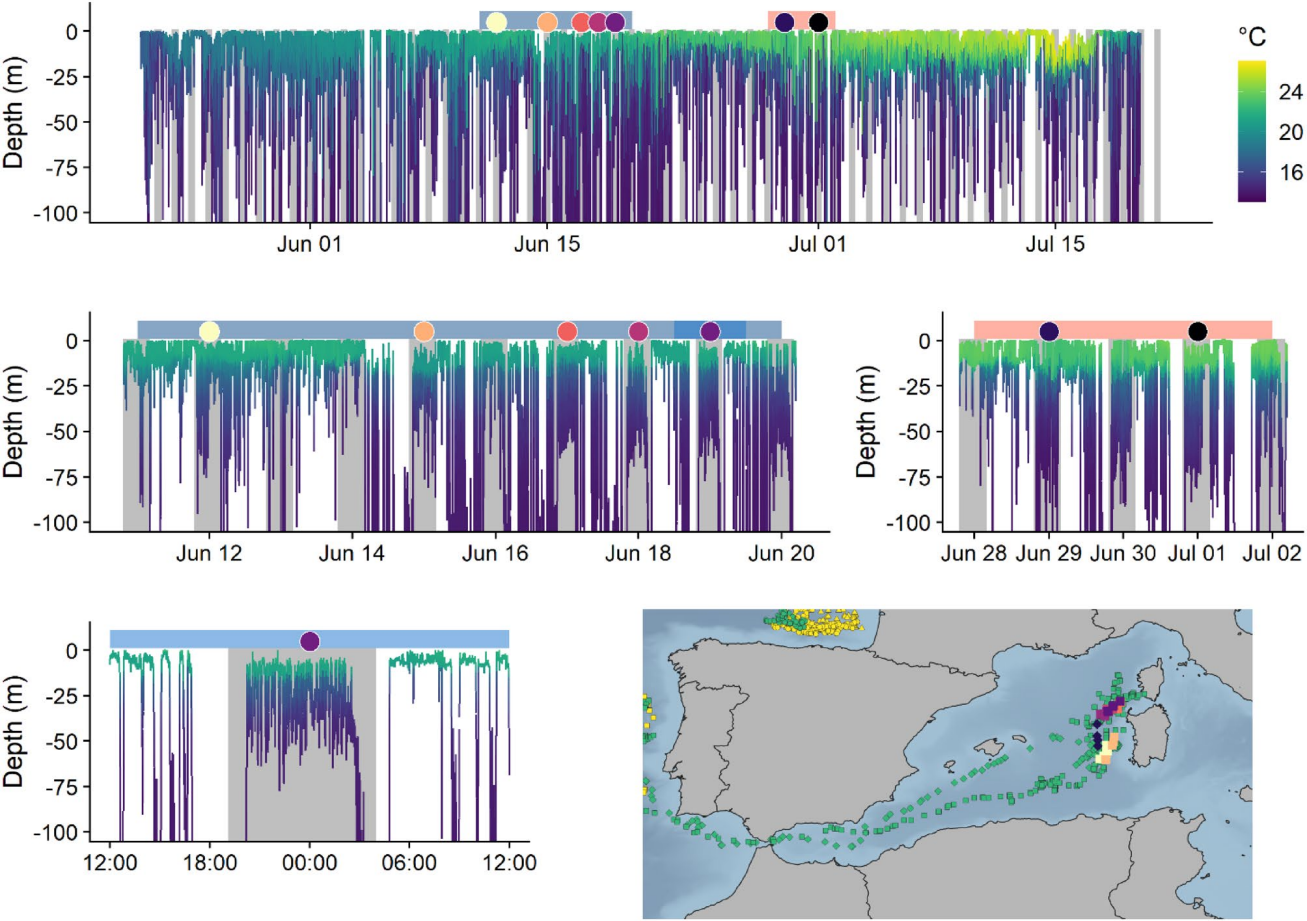
Putative spawning of a PSAT-tagged Atlantic bluefin tuna (34859), while in the Mediterranean Sea, in 2018. Upper panel shows depth and temperature during the entire period that the fish was in the Mediterranean Sea. Coloured bars above the graph in the top panel indicate the two periods presented in the middle panel. Coloured points indicate putative spawning events and corresponding locations are shown on the inserted map. A single putative spawning event is shown in detail in the bottom left panel. Bottom right panel show a subset of the modelled track overlaid with the dates spawning is identified with coloured dots (matching the colours in the upper panel).

Fish 34859 entered the Mediterranean Sea via the Gibraltar strait on 22 May 2018 and left again on 20 July 2018. By exploring the detailed temperature and depth profiles, and matching them with known spawning behaviour described in the literature^32,33^, 7 specific dates and locations for putative spawning events can be suggested (Fig. 4). These events occurred around mid-June (five events) and around late June/early July (two events). Temperatures experienced on these days often exceeded 20 °C. However, using the same approach on data from fish 34840 did not reveal any indications of spawning behaviour; temperatures and depth profiles did not match known spawning behaviour (i.e., nocturnal diving activities at temperatures above 20 °C).

## Discussion

Satellite tracking has yielded key information about the movements and behaviour of marine vertebrates in ways that were previously logistically impossible^34^. In the current study, we tagged the first 18 angler-caught ABFT in Skagerrak, and tracked their movements for up to one year. Despite the majority of tags detaching prematurely, our data provides new insights regarding the migration behaviour and habitat use of this species, both locally within the Nordic region and more widely throughout the northeast Atlantic and western Mediterranean Sea. Most fish (N = 9) left Skagerrak via the Norwegian Trench, heading north before exiting into the Atlantic. In addition, the two tags which remained deployed for approximately one full year showed a return migration into the Skagerrak from the northern North Sea and southern Norwegian Sea regions, re-entering north of the British Isles and through the Norwegian Trench. No fish exited or re-entered through the English Channel or the southern North Sea. These observations of entry/exit from the Skagerrak are similar to migration behaviour inferred from historical commercial fishery data in the region during the 1950s–1960s^16,19^. These historical records also demonstrated that some individuals migrated from the southern Norwegian Sea into the Skagerrak, Kattegat and Øresund, before leaving the area several weeks later, potentially indicating exploratory feeding on herring and mackerel, abundant in the area during this time of year. Our new tagging results confirm this behaviour among at least some of the ABFT migrating to these areas.

The migration patterns revealed by our tagging study exposes tuna entering and exiting the Skagerrak, Kattegat and Øresund to targeted exploitation by regional commercial fishing vessels. Presently, these vessels catch ABFT under a Norwegian quota (315 tonnes in 2021) but additional countries in the region may acquire a quota in the future. Moreover, the relatively narrow size distribution of tunas caught indicates that this migratory behaviour may only be performed by a limited number of year classes^35^, meaning that the continued long-term migration of ABFT to these waters is highly dependent on recruitment and survival of younger year classes. These younger year classes, perhaps once they reach a certain size, could then also undertake a migration to Skagerrak–Kattegat–Øresund. However, the combination of local exploitation pressures, and the presently limited number of year classes found in Skagerrak could result in ABFT migrating into Skagerrak–Kattegat–Øresund being a short-lived phenomenon if those year classes are subject to a large yearly fishing mortality (both regionally within the Nordic region, and more generally throughout the population range) and no younger year classes appear. Additionally, currently there is no scientifically-derived estimates of ABFT abundance for this region. We suggest to monitor the size distribution and abundance of ABFT in Scandinavian waters in the coming years to (1) confirm that visiting ABFT consist of only a few year classes, and clarify if younger year classes begin to appear, (2) evaluate how the numbers migrating to the region annually may change over time (e.g., under different levels of exploitation, or in relation to environmental factors).

While most of our tagged ABFT went north after exiting the Skagerrak, one individual turned south into the south-central North Sea before eventually leaving through the northern part of the North Sea. The region to which it migrated in the North Sea is congruent with earlier commercial catches and sightings in this region, including the Dogger Bank vicinity^15,16^. Although the exact routes that tagged individuals followed were not identical, no individuals used the shortest route to reach the Atlantic: from the Skagerrak through the North Sea to the English Channel, and further south to the Bay of Biscay and other southern regions. Migration along a northerly route probably reflects a trade-off between the potential for higher energetic gain from more abundant food and higher energy resources, and the longer migration distance. This could suggest that ABFT either follow the food, or simply follow the same route by which they came through learned behaviour.

Three tags remained attached long enough to explore long-term migration patterns and showed widely different behaviours. One fish crossed the Atlantic and utilized areas near the Grand Banks, crossing the ICCAT management boundary between the Western and Eastern stocks of ABFT (the 45° meridian), while the other two fish remained in the eastern Atlantic. The area west of Ireland, the Bay of Biscay and the area west of Portugal appear to be important feeding areas when the fish are not in Skagerrak or the Norwegian Sea. These results reflect interconnected seascapes for foraging through the NE Atlantic. Connecting foraging grounds off Ireland and the Bay of Biscay, which was previously suggested by Ref.^24^ is further corroborated by one of the fish tagged in this study, which passed over the Irish continental shelf when returning to Skagerrak in 2018.

### Depth and temperature use

Within ICES Area 3a, ABFT were predominantly roaming the upper water column, with most observations in the upper 100 m. However, some ABFT did dive to much deeper depths, with the maximum depth recorded being 520 m, showing that they can use the majority of the depth range available in the area (max. depth in the Norwegian Trench is app. 725 m, but represents a relatively small area). The behaviour likely reflects foraging, as ABFT were also observed by both the scientific tagging crews and the anglers to actively chase prey fish, like garfish and mackerel, at the surface during the tagging operations. The temperature ranges recorded varied between 7 and 17 °C. Both the depths and temperatures recorded are well within the thermal and depth limits reported in the literature for ABFT^36^.

### Spawning

ABFT have been shown to successfully spawn at temperatures above 20 °C at night^30,31^, and to display a distinct dive pattern thought to represent courtship and spawning behaviour^29^. When matching this described behaviour with the data from fish 34859 in the Mediterranean Sea, almost identical behavioural patterns were detected on specific days (Fig. 4). In total, seven days aligned with temperatures above 20 °C and oscillatory movement past the thermocline. All detected spawning events occurred west of Sardinia, where fishing for mature ABFT has been conducted for centuries^37^.

In light of the recently proposed third spawning area in the Slope Sea of northeast United States^38^ and other proposed areas outside the Mediterranean^19^, it is relevant to look for similar temperature and behavioural patterns for fish 34840, which did not enter the Mediterranean Sea, and instead stayed in the eastern Atlantic. We found that this fish did not display a similar oscillatory behaviour, and the temperature experienced during the alleged spawning period (June–July) was above 20 °C only once (20.4 °C on 11 July). In this period, the fish was on the continental shelf west of Ireland, likely feeding and not spawning. Due to the size of the fish (247 cm CFL), reflecting a likely age of 14–16 years (matching the strong 2003 cohort), and the assumption that all eastern ABFT above five years and western ABFT above eight years are mature, we find it unlikely that this fish was immature. As such, these observations may suggest that this fish skipped spawning in 2018. Fish 34861 surfaced on 25 April and the tag was not recovered. The transmitted data does not allow for a detailed analysis of potential spawning behaviour for this fish. It did however, display 6 days where maximum temperatures from the transmitted dataset reached 20 °C (observations from 15. March to 20 April, with temperatures ranging from 20 to 20.6 °C). Given the lack of detailed behaviour and the fact that this time is well outside the normal spawning time for Mediterranean ABFT, we propose that this ABFT did not spawn in that period. However, the documentation of spawning depends on the general applicability of the temperature limits and nightly spawning behaviour^30,31^. More studies documenting spawning behaviour will be needed to corroborate if this pattern is consistent among locations and stocks. We also suggest more studies with longer lasting tags to elucidate if skipped spawning is a common behaviour and if fish skip one or more consecutive spawning seasons. Skipped spawning has been demonstrated in many fish species, including both freshwater and marine fish^39^, and likely reflects physiological condition^40^. If a considerable proportion of the adult population skips spawning every season, current population models, which assume annual spawning by all adult fish, should be modified to more accurately reflect population egg production and reproductive output. Current population modelling may be even further challenged if the proportion of adults that skip spawning varies over time, perhaps depending on environmental conditions. However, we acknowledge that only one of two fish followed through the spawning season appeared to skip spawning, and therefore caution against broad general interpretations. More studies are needed to verify that skipped spawning is a common behaviour, and if so, to estimate just how common that behaviour is.

### Return migration

In exploited fish populations, large adults are hypothesized to be important components of the spawning population because they contribute more to recruitment than smaller individuals due to a variety of maternal effects including higher fecundity, better quality of eggs and differences in spawning behaviour (e.g. time, location)^41^. Although such effects remain to be documented for ABFT, it may be prudent to conserve these large individuals as a precautionary measure, to maximize their potential contributions to reproduction and recruitment.

In order to protect these fish, new knowledge about their movements and distribution is required. Data from ABFT deployed with long-term electronic tags suggests that after spawning in the Gulf of Mexico, the fish return to the feeding grounds where they were initially tagged, indicating a return feeding migration^7^. The same has been observed more recently from ABFT tagged in Ireland^24^, and other large highly migratory fish species (e.g., swordfish, *Xiphias gladius*^42^). In the current study, both ABFT that retained the tag for one year also returned to the same area, suggesting a similar seasonal return feeding migration. We also note that ABFT appeared to perform recurrent visits to the Norwegian Sea, Ireland and the Bay of Biscay on their way from Nordic waters and upon their return to the latter. Hence, we hypothesize that large ABFT in Nordic waters generally return to the same feeding area the following year, given suitable habitat features (e.g., food and temperature conditions), and follow a similar migration route as they do so. More studies are nonetheless needed to confirm this hypothesis, given few long-term deployments in the current study. For a deeper understanding of behavioural repeatability, and if/when shifts in the behaviour occur, it will be necessary to follow the same fish over multiple years. Such studies would also act as a highly valuable indicator of survival, independent of stock assessment-derived mortality estimates, and could be used to estimate the local abundance of larger ABFT^43^. Thus, a promising avenue for future research would be to deploy long-lasting (> 5 to 10 years) acoustic tags and use existing infrastructure from networks such as the European Tracking Network to track these large fish over the next decade^44^. Given that ABFT appear to return to the area annually, we suggest that Skagerrak is a promising area for the future deployment and retrieval of PSATs and other long-lasting tags, because of the relatively easy access to locate and recover detached floating tags, given that the area is reachable from land within a few hours by boat. Retrieving PSATs that have detached from animals enables scientists to access full datasets (in the present case with 5 s resolution, rather than the much coarser and variable resolution typically transmitted). This much higher resolution enables much more detailed analysis, as shown in our analysis of spawning behaviour. Additionally, floating Pop-off Data Storage Tags (PDST) tags may also be a prominent and less costly avenue forward as the geographical region is densely populated and contains many sandy beaches and highly visited coastal areas, giving ample opportunity for tag recovery. Previous studies with floating DSTs in this area have shown remarkably high return rates^45^.

The evidence that ABFT have returned to Nordic waters following many years of rarity or absence, and our findings that at least *some* individuals return to the same site for feeding in consecutive years, raises new questions about the mechanisms that underlie habitat discovery—or the return to previously used habitats—by highly migratory fish species. How individuals or entire schools have discovered this region again as a suitable feeding area after an absence of more than 50 years is unclear. In light of the positive stock development in the last 1–2 decades^22^ and modelling studies showing suitable habitat in the area^46^, density-dependent foraging and exploratory behaviour for new feeding areas may be a prominent hypothesis for their return, potentially accompanied by complex social learning interactions among individuals within the population^47,48^. New tagging data which documents the use of new or formerly occupied habitats will be essential for understanding these processes and how they might be affected by human pressures (e.g., exploitation, climate change). Such data can help to parameterize and validate advanced conceptual models of group movement behaviour, collective memory and habitat use^49–51^, as well as to inform modern stock assessment models used for management.

### Tag deployment

Following recommendations from experienced taggers previously operating in the Mediterranean, most fish were tagged in the water alongside the boat. All these tags surfaced prematurely, while two (out of three) tags deployed on tunas brought on board the tagging boat surfaced after approximately one year. Depending on the conditions at sea, tagging along the side of the boat may not be as precise as on-board tagging, and the quality of the tag anchoring cannot be properly assessed. We therefore suggest that tagging on-board a boat is superior to tagging in the water alongside the boat for the deployment of long-lasting tags. This was also suggested in Ref.^24^. Furthermore, on-board tagging makes biological sampling fast and feasible, as opposed to tagging in the water alongside the boat. However, our advice is limited by a small sample size, making it difficult to draw formal conclusions; more studies are necessary to assess the best method to tag large ABFT.

## Conclusion

Large ABFT have returned to Skagerrak in recent years following decades of absence. Tagging with advanced electronic tags (PSAT) showed that the ABFT visiting Skagerrak exit via the Norwegian Trench. Tracking data demonstrated individual differences in behaviour, with one fish moving into the Mediterranean to spawn, one fish staying exclusively in the Atlantic without spawning, and one fish moving across management boundaries. Spawning behaviour was shown in one ABFT, on seven different dates west of Sardinia, Italy. Two fish returned to the area of tagging after approximately 1 year, suggesting a return feeding migration. This return enables repeated opportunities to study large ABFT over multiple years, and potential targeted retrieval of surfaced tags, though more studies are needed to confirm the present results. The observations may have important implications for management because (1) tunas that come into Nordic waters may be vulnerable to area-specific exploitation, and (2) they challenge the assumption of consecutive spawning in ABFT population models.

## Data Availability

Data available upon request from the corresponding author.
